# Supplementary figures and images for: Dietary fiber intake and cognitive impairment in older patients with chronic kidney disease in the United States: A cross-sectional study
Source: PLoS One. 2023 Oct 4;18(10):e0291690. doi: 10.1371/journal.pone.0291690 (PMC10550150; doi:10.1371/journal.pone.0291690)

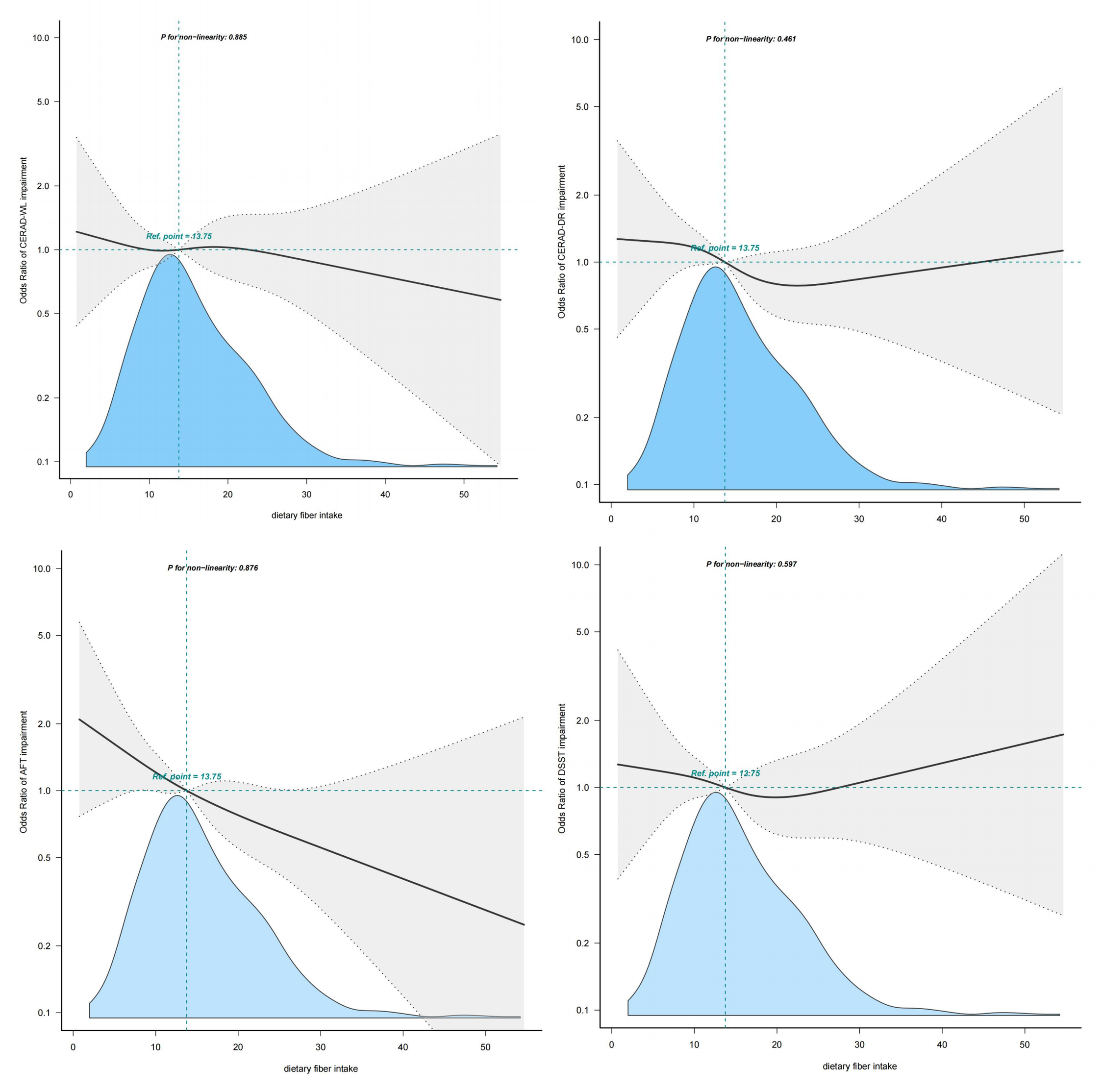

Supplement: S1 Fig — Solid and dashed lines represent the predicted value and 95% confidence intervals. The models were fully adjusted by age, sex, ethnicity, education, marital status, smoking, body mass index, hypertension, diabetes, coronary heart disease, stroke, malignancy, depression, sleep disorder, UACR, eGFR, hemoglobin, albumin, blood urea nitrogen, creatinine, uric acid, dietary energy, dietary protein, and dietary carbohydrate. Abbreviations: CKD, chronic kidney disease; UACR, urinary albumin:creatinine ratio; eGFR, estimated glomerular filtration rate; CERAD-WL, Consortium to Establish a Registry for Alzheimer’s Disease Word Learning; CERAD-DR, Consortium to Establish a Registry for Alzheimer’s Disease Delayed Recall; AFT, Animal Fluency test; DSST, Digit Symbol Substitution test. (TIF) [file pone.0291690.s001.tif]
