# Supplementary material for: Dietary fiber intake and cognitive impairment in older patients with chronic kidney disease in the United States: A cross-sectional study
Source: PLoS One. 2023 Oct 4;18(10):e0291690. doi: 10.1371/journal.pone.0291690 (PMC10550150; doi:10.1371/journal.pone.0291690)
Supplement: S2 Table — The logistic regression models were adjusted by age, sex, ethnicity, education, marital status, smoking, body mass index, hypertension, diabetes, coronary heart disease, stroke, malignancy, depression, sleep disorder, UACR, eGFR, hemoglobin, albumin, blood urea nitrogen, creatinine, uric acid, dietary energy, dietary protein, and dietary carbohydrate. Abbreviations: CKD, chronic kidney disease; NHANES, National Health and Nutrition Examination Survey; UACR, urinary albumin:creatinine ratio; eGFR, estimated glomerular filtration rate; CERAD-WL, Consortium to Establish a Registry for Alzheimer’s Disease Word Learning; CERAD-DR, Consortium to Establish a Registry for Alzheimer’s Disease Delayed Recall; AFT, Animal Fluency test; DSST, Digit Symbol Substitution test. (DOCX) [file pone.0291690.s003.docx]

**S2 Table CKD and cognitive function impairment by levels of fiber intake among older adults after excluding CKD patients receiving dialysis treatment in the US , NHANES 2011–2014.**

| Cognitive tests | Total | | Low fiber intake  (≤25g/day)^﹡^ | | High fiber intake  (>25g/day)^﹡^ | | P for interaction |
| --- | --- | --- | --- | --- | --- | --- | --- |
|  | OR(95%CI) | P-value | OR(95%CI) | P-value | OR(95%CI) | P-value |  |
| CERAD-WL |  |  |  |  |  |  | 0.927 |
| Non-CKD | 1(Ref) |  | 1(Ref) |  | 1(Ref) |  |  |
| CKD | 1.39(1.09~1.78) | 0.009 | 1.39(1.07~1.8) | 0.014 | 1.39(0.62~3.15) | 0.425 |  |
| CERAD-DR |  |  |  |  |  |  | 0.827 |
| Non-CKD | 1(Ref) |  | 1(Ref) |  | 1(Ref) |  |  |
| CKD | 1.25 (0.98~1.6) | 0.067 | 1.21(0.93~1.56) | 0.153 | 1.85(0.83~4.13) | 0.131 |  |
| AFT |  |  |  |  |  |  | 0.260 |
| Non-CKD | 1(Ref) |  | 1(Ref) |  | 1(Ref) |  |  |
| CKD | 1.22(0.96~1.56) | 0.109 | 1.28(0.99~1.65) | 0.065 | 0.91(0.38~2.21) | 0.843 |  |
| DSST |  |  |  |  |  |  | 0.955 |
| Non-CKD | 1(Ref) |  | 1(Ref) |  | 1(Ref) |  |  |
| CKD | 1.42(1.07~1.88) | 0.015 | 1.44(1.07~1.94) | 0.017 | 1.7 (0.61~4.71) | 0.308 |  |
