# Supplementary material for: Dietary fiber intake and cognitive impairment in older patients with chronic kidney disease in the United States: A cross-sectional study
Source: PLoS One. 2023 Oct 4;18(10):e0291690. doi: 10.1371/journal.pone.0291690 (PMC10550150; doi:10.1371/journal.pone.0291690)
Supplement: Attachment — Submitted filename: Response to Reviewers.docx [file pone.0291690.s005.docx]

**Reviewer #1**:

Abstract: the sentence "A total of 2461 older adults were included, of with 32% suffered from CKD" should be rephrased.
Response：Thank you for pointing it out. We have revised the sentence.

Page 2, lines 29

“A total of 2461 older adults were included, with 32% who suffered from CKD.”

The language needs to be futher polished.
Response：Thank you for your comment. The manuscript has been edited by a professional language editing service and the certificate was provided. If required, we would like further polish the manuscript.

The sentence "Previous studies suggested that protein and carbohydrate intakes, which are the main dietary components, were associated with cognition [13,14]." should be rewritten for clarity.
Response：Thank you for pointing it out. We have revised the sentence.

Page 8, lines 126-127

“As the main dietary components, protein and carbohydrate intakes were reported to associated with cognition in previous studies.”

Line 79, the word "peportedly" is inappropriately used.

Response：Thank you for pointing it out. We have corrected the word.

**Reviewer #2**: Overall, I have no major criticisms on this ms; however, restricted cubic splines should be mentioned in the methods and the results should be provided in the text.

Response：Thank you for your valuable comment. Restricted cubic splines were performed, and the results and figures have been added to the article.
